# Rituximab in non-systemic vasculitic neuropathy: a single-center experience

**DOI:** 10.1007/s00415-024-12378-1

**Published:** 2024-04-24

**Authors:** Felix Kohle, Gilbert Wunderlich, Gereon R. Fink, Michael Schroeter, Helmar C. Lehmann, Christian Schneider

**Affiliations:** 1 Department of Neurology, Faculty of Medicine, University of Cologne and University Hospital Cologne, Cologne, Germany; 2 Center for Rare Diseases, Faculty of Medicine and University Hospital, University of Cologne, Cologne, Germany; 3 Cognitive Neuroscience, Research Center Juelich, Institute of Neuroscience and Medicine (INM-3), Juelich, Germany; 4 Department of Neurology, Hospital Leverkusen, Leverkusen, Germany; 5 Department of Neurology, St. Katharinen Hospital, Frechen, Germany

**Keywords:** Polyneuropathies, Vasculitis, Drug repurposing, Antibodies, b-lymphocytes, Peripheral nervous system

## Abstract

**Objectives:**

This case series reports clinical features and outcome of four patients with non-systemic vasculitic neuropathy (NSVN) treated with the anti-CD20 agent rituximab.

**Methods:**

Clinical, electrophysiological and biopsy data were retrospectively obtained and evaluated. Only patients with pathological definite or probable NSVN were included. Extensive clinical and laboratory work-up excluded systemic vasculitis. Follow-up data for at least 12 months and up to five years is provided. Outcome of the patients was assessed using the MRC-Sum Score, Prineas Score and Neurological Symptom Score.

**Results:**

Two of four patients treated with rituximab achieved disease remission and one patient remained stable under anti-CD20 therapy after a required treatment switch due to toxic side effects of cyclophosphamide. One patient deteriorated under rituximab induction. Rituximab was well tolerated in all patients.

**Discussion:**

Anti-CD20 therapy might be an alternative in NSVN patients requiring further treatment escalation or treatment switch due to side effects of corticosteroids or cyclophosphamide.

## Introduction

Several new treatment options with improved side effect profiles compared to conventional chemotherapeutics for neurological autoimmune disorders, such as multiple sclerosis, but also for rare neuromuscular disorders, such as myasthenia gravis, were approved in recent years, leading to a shift of the therapeutic landscape. However, for immune-mediated neuropathies, these progresses are still lacking [1]. There are several reasons for this, e.g., the heterogeneity and the rarity of the diseases. Non-systemic vasculitic neuropathy (NSVN) is a rare immune neuropathy, with distally attenuated asymmetric motor and sensory deficits and mainly electrophysiological characteristics of axonal nerve damage [2]. NSVN responds to immunomodulatory treatment, but larger prospective randomized trials are missing and treatment regimens are based on retrospective cohort studies [3–5]. According to guideline recommendations, corticosteroids alone or in combination with cyclophosphamide, azathioprine and methotrexate should be used. For severe cases, cyclophosphamide should be given to achieve clinical remission in the patients [3]. However, long-term treatment with cyclophosphamide has relevant dosage-related and treatment limiting myelotoxic side effects. Other escalating therapies option are currently not available. Extrapolation from randomized-controlled trials (RCTs) in small and medium vessel primary systemic vasculitides indicate that intravenous immunoglobulin, plasma exchange, and rituximab might be treatment options for NSVN patients [2]. Here, we report the clinical outcome and tolerability of rituximab treatment in four NVSN patients.

## Methods

All patients were diagnosed with NSVN in the Department of Neurology at the University Hospital of Cologne between 2011 and 2018. Clinical and treatment data, including neurophysiological examinations and neuropathological results of nerve biopsies, were retrieved retrospectively from medical reports from first admission to December 2023 (approved by the local ethic committee, 21-1330). All four patients had been diagnosed with a pathologically confirmed definite (*n* = 1) or probable (*n* = 3) NSVN according to guideline [3]. An experienced neuropathologist analyzed all biopsy specimens. No patient had signs of a systemic vasculitis by clinical or laboratory measures, including ANCA-antibodies, serum electrophoresis, differential blood count, C-reactive protein, erythrocyte sedimentation rate, and cerebrospinal fluid analysis. Other causes of neuropathies had been excluded due to their individual history, presentation, and laboratory tests (e.g. immunofixation). As outcome variables, changes of sensorimotor symptoms evaluated by the Medical Research Council (MRC) sum score [6], Prineas Score [7], and Neurological Symptom Score (NSS) [8], from 12 months prior to rituximab induction, baseline (rituximab induction) and to follow-up (every 12 months) were included. All three scores have been used to quantify changes in motor symptoms [4] and in sensory symptoms [5] in NSVN.

Descriptive data are presented with GraphPad Prism, version 10.0.3 for Windows, GraphPad Software, Boston, Massachusetts USA.

## Results

### Clinical, electrophysiological and biopsy characteristics

Mean age of the patients was 57.72 years at the time of the data curation with two male and two female patients. Diabetes mellitus type 2 was reported in two patients as a relevant comorbidity. The clinical presentation of NSVN ranged from sensorimotor deficits in three patients with a symmetric or asymmetric pattern to a pure sensory presentation in one patient. Sensorimotor affection led to impaired walking in all patients, and neuropathic pain was reported in every patient. All four biopsies showed vessel-wall infiltration of T-cells, infiltration of leucocytes and epineural T-cells, leading to axonal nerve fiber loss in all patients. Notably, B-cells were detected in just one biopsy (Patient 4). Further clinical and electrophysiological data are presented in Table 1.

**Table 1:** Clinical, electrophysiological (compound muscle action potential (CMAP), sensory nerve action potential (SNAP) and nerve conduction velocity (NCV)) and biopsy characteristics of the patients treated with rituximab

Demographic data	Age at diagnosis (range in years, mean)	Gender (female: male)	Age at end of follow-up (range in years, mean)	Relevant comorbidities
	45.52–74.71, 57.72	2:2	48.27–80.67, 65	Diabetes mellitus type 2 (n = 2)

Symptoms	Affected systems (sensorimotor, pure sensory, pure motor)	Symptoms distribution (asymmetric, symmetric)	Walking (normal, impaired, walker, wheel-chair)	Neuropathic pain (present, not present)
Patient 1	Sensorimotor	Asymmetric	Impaired	Present
Patient 2	Sensorimotor	Symmetric	Walker	Present
Patient 3	Pure sensory	Symmetric	Normal	Present
Patient 4	Sensorimotor	Asymmetric	Impaired	Present

Biopsy	Pathological vasculitic neuropathy (yes, no)	T- and B-cells distributions	Macrophages	Neuronal damage (axonal, demyelinating)
Patient 1	Probable Vessel-wall infiltrating T-cells Prominent active axonal degeneration	CD4 positive T-cells No B-cells	Yes	Mainly axonal
Patient 2	Probable Vessel-wall infiltrating T-cells Hemosiderin deposits	CD4 and CD8 positive T-cells No B-cells	Yes	Mainly axonal, some demyelination
Patient 3	Definite Vessel-wall infiltrating T-cells Adventitial/periadventitial fibrosis	More CD4 than CD8 positive T-cells No B-cells	Yes	Mainly axonal, some demyelination
Patient 4	Probable Vessel-wall infiltrating T-cells Asymmetric/multifocal nerve fiber loss or degeneration	More CD4 than CD8 positive T-cells Few B-cells	Yes	Mainly axonal

Nerve conduction studies at rituximab start	N. tibialis (CMAP in mV. mNCV in m/s)	N. suralis (SNAP in µV. sNCV in m/s)	N. ulnaris—motor (CMAP in mV. mNCV in m/s)	N. ulnaris—sensory (SNAP in µV. sNCV in m/s)
Patient 1	n.a	n.a	n.a	n.a
Patient 2	Right: **0.1 mV,** 54 m/s	Right:** no response** Left:** no response**	Right: **7.3 mV,** 61 m/s	Right:** 3 µV, 33 m/s**
Patient 3	Right: 20.6 mV, 47 m/s	Right:** no response** Left:** no response**	n.a	n.a
Patient 4	Right:** no response**	Right:** no response**	Right: 11.5 mV, 60 m/s	Right: **9 µV, 42 m/s**

Nerve conduction studies at 12 months follow-up	N. tibialis (CMAP in mV. mNCV in m/s)	N. suralis (SNAP in µV. sNCV in m/s)	N. ulnaris—motor (CMAP in mV. mNCV in m/s)	N. ulnaris—sensory (SNAP in µV. sNCV in m/s)
Patient 1	n.a	n.a	n.a	n.a
Patient 2	n.a	n.a	n.a	n.a
Patient 3	Right: 21.5 mV, 43 m/s	Right: **3 µV**, 43 m/s	Right: 12.4 mV, 58 m/s	Right: 13 µV, 48 m/s
Patient 4	Right:** 0.1 mV, 38 m/s**	Right:** no response**	Right: 11.2 mV, 47 m/s	Right 17 µV, 53 m/s

Missing data is indicated by n.a. (not available)

### Treatment characteristics and outcome

Overall disease duration at the first cycle of rituximab differed widely between the four patients (from 0.75 years to 11.69 years, mean 4.44 years). Prior to escalating the therapy to rituximab, all patients received corticosteroids and cyclophosphamide, and in one patient intravenous immunoglobulins were tried (Table 2). Treatment switch to rituximab was initiated due to disease progression in three patients. Patient 3 suffered from nausea under cyclophosphamide therapy and patient 4 was stable under cyclophosphamide, but overall high cumulative dosages and relevant side effects (hemorrhagic cystitis, basal cell carcinoma) necessitated a treatment switch to rituximab. Rituximab dosages were well-tolerated, with no reported side effects. No concomitant oral corticosteroids during induction and maintenance therapy were given. For treatment induction, rituximab 500 mg was given intravenously twice and then given once every 6–9 months for maintenance therapy as described in the MAINRITSAN-trial for ANCA-associated vasculitides [9]. Before rituximab infusions, 250 mg of methyl-prednisolone and an H1-antagonist were given intravenously.

**Table 2:** Treatment regimens prior to rituximab induction, disease duration at first cycle and reasons for treatment switch to rituximab

Prior treatments	Corticosteroids (oral)	Corticosteroids (pulsed intravenous)	Immunoglobulins (intravenous)	Cyclophosphamide (cumulative dosage in mg, number of dosages)
Patient 1	Yes	Yes	No	10,474 mg, *n* = 7
Patient 2	Yes	Yes	Yes	10,474 mg, *n* = 7
Patient 3	Yes	Yes	No	4316 mg, *n* = 4
Patient 4	Yes	No	No	63,339 mg, *n* = 45

Rituximab treatment	Disease duration at first cycle of rituximab (years)	Reasons for treatment switch	Received rituximab cycles	Response
Patient 1	0.75	Progress	1	No, disease progression within the first 2 months, then treated with immunoadsorption cycles and pulsed corticosteroids
Patient 2	3.05	Progress	1	Yes
Patient 3	2.28	Progress nausea	9	Yes
Patient 4	11.69	Hemorrhagic cystitis, basal cell carcinoma	3	Remained stable

**Fig. 1:**
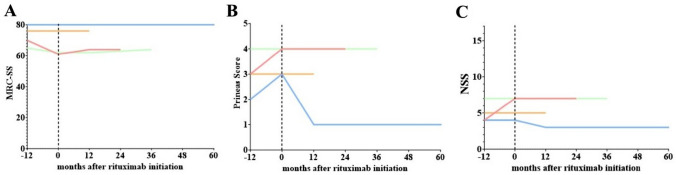
Outcome for the patients measured by Medical Research Council sumscore (MRC-SS, **A**), Prineas Score (**B**) and Neurological Symptom Score (NSS,** C**). Color coding for all graphs: red = patient 1, green = patient 2, blue = patient 3 and orange = patient 4

Follow-up data for the first 12 months after the first cycle of rituximab were retrieved in all patients and in one patient up for to 60 months (mean 33 months). Two patients received just one cycle of rituximab. Patient 1 deteriorated clinically within the first two months and was switched to pulsed corticosteroids and two cycles of immunoadsorption before stabilizing within the first year after rituximab treatment. In patient 2 partial remission was achieved without any further immunomodulatory treatment while in patient 3 disease remission was achieved with continuous rituximab treatment according to guideline [3], as also depicted by MRC-SS, Prineas-Score and NSS (Fig. 1). Patient 4 remained stable after treatment switch to rituximab.

## Discussion

Our case series displayed our single center experience for using the B-cell depleting agent rituximab in NSVN patients. NSVN is believed to be a mainly T-cell-mediated neuropathy, and guidelines recommend the use of the more T-cell directed immunosuppressants, such as corticosteroids and cyclophosphamide as first- and second-line treatments [3]. Accordingly, nerve biopsy specimen of NSVN patients in a larger cohort showed only few CD20 positive lymphocytes in only 50% of the biopsies [10]. However, B-cells regulate autoimmunity mostly not in a compartment specific pattern and localized pathological descriptions given by biopsies therefor offer only limited insights [11]. B-cell depletion impairs CD4+ T-cell activation and clonal expansion. It has been shown that CD4+ activation before tissue migration occurs in the regional lymph nodes [12, 13]. Further, complement activation in NSVN biopsies was detected in another study, for which (auto-)antibody-binding is critical for the classical pathway [14]. B-cell depletion is highly effective in preventing further relapses in other mainly T-cell-mediated disease, such as multiple sclerosis, highlighting the complex interplay and pivotal role of B-cells in sustaining autoinflammation. Monoclonal antibodies, in this case rituximab to target specifically B-cells, have several advantages compared to conventional chemotherapeutics, e.g. cyclophosphamide and azathioprine. Long-term dosage dependent cancerous effects are not observed and the drugs often show a favorable side effect profile, as shown by real-world data of on- and off-label use of anti-CD20 agents for autoimmune neurological diseases, such as multiple sclerosis [15]. For corticosteroids, long-term side effects of higher dosage are most-often therapy limiting. For many rheumatological and neurological autoimmune diseases, monoclonal antibodies replaced conventional chemotherapeutic drugs as first-line and escalation therapy. Side-effects were also a limiting factor for our patients. One patient suffering from short-term side effects of cyclophosphamide. The fourth patient was stable under cyclophosphamide, but long-term and dosage dependent side effects required a therapy switch. The observed side effect profile of rituximab was beneficial in our patients without reporting of any serious infections.

Regarding other cases of NSVN treated with rituximab, we identified two case reports with two patients each. In the first report, remission with a co-induction therapy of rituximab and oral corticosteroids was achieved [16]. In the second case report, clinical improvement with an escalation therapy of both, intravenous immunoglobulins and rituximab was successfully used in two patients with NSVN [17]. In both case reports, extensive clinical and histopathological characterization and long-term follow-up data are missing. We could not identify any further case reports or case series reporting the use of rituximab or another anti-CD20 agent for NSVN, however, extrapolation from RCTs of the small-to-medium vessel primary systemic vasculitides indicate a potential, but unproven benefit for rituximab [2, 3].

There are some uncertainties regarding the therapy scheme of rituximab. For multiple sclerosis, some centers prefer higher induction dosages of 2 × 1 g and a maintenance dose of 1 g every 6–12 months [18], while in Europe the effective use of much lower dosages was reported [19]. In hematological disorders the dosage is usually calculated based on body surface (375 mg/m^2^ four times in the first months, followed by maintenance doses every six month) [20]. It is possible that suboptimal dosing was the reason for no observed clinical response to rituximab in patient 1. B-cell lysis could be another reason for the observed deterioration of the patient that could be addressed with corticosteroid bridging [21].

This study has clear limitations. First, the overall number of four patients is too small to draw relevant conclusions of the efficacy and safety profile of rituximab in NVSN. Second, even though all patients had pathological definite or probable diagnosis of NSVN, two patients also had diabetes mellitus type 2, possibly confounding disease progression data. Third, although hepatitis C infection as the main cause of cryoglobulinemic vasculitis was excluded in all patients and serum C3 levels used as surrogate marker were normal, no highly sensitive and specific diagnostic immunofixation or -electrophoresis was performed to definitively exclude cryoglobulinemic vasculitis [22].

Nevertheless, this case series offer valuable data to guide neurologist for escalation or therapy switch in NSVN and suggest that the use of rituximab might be treatment option for severe NSVN cases. Treatment alternatives in rare autoimmune neuropathies such as NSVN are urgently needed to either halt disease progression or minimize long-term and short-term side effects of other immunosuppressants. Drug repurposing might be a valuable tool as studies for these rare diseases due to their heterogeneity are hard to conduct [1].

## Data Availability

The data are not publicly available due to privacy or ethical restrictions. Anonymized data not published within this article will be made available upon reasonable request from any qualified investigator.

## Abbreviations

CMAP: Compound muscle action potential
MRC-SS: Medical Research Council sum score
NCV: Nerve conduction velocity
NSS: Neurological symptom score
NSVN: Non-systemic vasculitic neuropathy
RCT: Randomized-controlled trial
SNAP: Sensory nerve action potential
